# Perceived Neighborhood Disorder, Social Cohesion, and Depressive Symptoms among Spousal Caregivers

**DOI:** 10.1093/geroni/igab046.3719

**Published:** 2021-12-17

**Authors:** Yeon Jin Choi, Jennifer Ailshire

**Affiliations:** University of Southern California, Los Angeles, California, United States

## Abstract

Most prior research on caregivers’ mental health focused on individual or household factors, we know much less about the influence of neighborhood factors on mental health of spousal caregivers. The current study fills the gap in our knowledge by examining the association of neighborhood characteristics (i.e., perceived neighborhood disorder and neighborhood social cohesion) and depressive symptoms among spousal caregivers. We used data from 2006 to 2016 waves of the Health and Retirement Study, which includes 2,362 spousal caregivers. Negative binomial regression models were estimated to examine the association of perceived neighborhood disorder and neighborhood social cohesion with depressive symptoms. A greater perceived neighborhood disorder was associated with higher CES-D scores, which indicates more depressive symptoms. On the other hand, a higher level of neighborhood social cohesion was associated with lower CES-D scores. When they were included in the same model, the association between neighborhood disorder and depression disappeared, while respondents who reported higher levels of neighborhood social cohesion continue to exhibit lower CES-D scores than those lived in less cohesive neighborhoods. This study highlights the importance of neighborhood contexts in understanding caregivers’ well-being. Findings of this study suggest that neighborhood social cohesion may attenuate the negative effects of neighborhood disorder. Therefore, enhancing positive characteristics of the neighborhood may promote well-being of spousal caregivers.

